# Distinct alterations of CD68^+^CD163^+^ M2-like macrophages and myeloid-derived suppressor cells in newly diagnosed primary immune thrombocytopenia with or without CR after high-dose dexamethasone treatment

**DOI:** 10.1186/s12967-018-1424-8

**Published:** 2018-03-02

**Authors:** Xia Shao, Boting Wu, Luya Cheng, Feng Li, Yanxia Zhan, Chanjuan Liu, Lili Ji, Zhihui Min, Yang Ke, Lihua Sun, Hao Chen, Yunfeng Cheng

**Affiliations:** 1 Department of Hematology, Zhongshan Hospital Fudan University, Shanghai, 200032 China; 2 Department of Transfusion Medicine, Zhongshan Hospital Fudan University, Shanghai, 200032 China; 3 Department of Hematology, Zhongshan Hospital Qingpu Branch, Fudan University, Shanghai, 201700 China; 4 Institute of Clinical Science, Department of Hematology, Zhongshan Hospital, Fudan University, 180 Fenglin Rd, Shanghai, 200032 China; 5 Shanghai Institute of Clinical Bioinformatics, Fudan University Center for Clinical Bioinformatics, Shanghai, 200032 China; 6 Department of Thoracic Surgery, Zhongshan Hospital Qingpu Branch, Fudan University, Shanghai, 201700 China

**Keywords:** Immune thrombocytopenia, M2-like macrophages, MDSCs, Dexamethasone

## Abstract

**Background:**

Although impaired myeloid-derived suppressor cells (MDSCs) recently have been studied in immune thrombocytopenia (ITP), another myeloid-derived cell population signified as M2 macrophages has not been investigated properly in ITP patients. In the present study, we intended to determine the features of circulating M2-like macrophages, to examine its relationship with MDSCs, and to explore their prognostic values in ITP.

**Methods:**

Peripheral blood mononuclear cells from healthy controls and primary ITP patients were isolated to test the circulating M2-like macrophages and MDSCs. The circulating M2-like macrophage population defined as CD68^+^CD163^+^ and circulating MDSC population as CD11b^+^CD33^+^HLA-DR^−^ were determined by flow cytometry. Plasma inflammatory cytokines were measured by multiplex ELISA.

**Results:**

The percentages of MDSCs were found to be expanded in newly diagnosed patients of ITP, especially among those of the complete response (CR) group (*p* < 0.0001). Positive linear correlation was verified between percentages of M2-like macrophages and MDSCs. The same correlation was also determined in the CR group. After treatment, the percentages of M2-like macrophages and MDSCs were both increased significantly in CR group, while those patients among the PR + NR group manifested a significant numeric decrease of MDSCs but only a moderate decrease in M2-like macrophages. MIP-1α/CCL3 was negatively correlated with M2-like macrophages while MCP-1 possessed a positive correlation with M2-like macrophages, eotaxin-1/CCL11 was negatively correlated with MDSCs and interleukin-1β (IL-1β) was found to be negatively correlated with both M2-like macrophages and MDSCs.

**Conclusions:**

The present findings indicated critical roles of both circulating M2-like macrophages and MDSCs in ITP. The positive correlation between them might be related to inflammatory factors-mediated bidirectional interactions or partially due to their similar background patterns during differentiation. MIP-1α/CCL3, MCP-1, eotaxin-1/CCL11 and IL-1β might play a critical role in the expansion of both M2 macrophages and MDSCs population in ITP patients, which deserves further investigation.

**Electronic supplementary material:**

The online version of this article (10.1186/s12967-018-1424-8) contains supplementary material, which is available to authorized users.

## Background

Immune thrombocytopenia (ITP) is an acquired autoimmune hemorrhagic disorder, characterized by immune-mediated platelet destruction and impaired megakaryocyte maturation [1]. High-dose dexamethasone (HD-DXM), an appropriate first-line therapy for adult patients with ITP [2], has achieved a satisfying initial response rate up to 85% [3]. Clinical investigations for the pathogenesis of ITP have been largely focused on the aberrations related to adaptive immunity including platelet autoantibodies [4], T cell clonality and T helper differentiation [5]. Recently, the role of innate immunity and myeloid-derived immune modulator cells have been recognized in the pathogenesis of ITP [6, 7].

Myeloid-derived suppressor cells (MDSCs), generally recognized as circulating immature myeloid cells with remarkable immunosuppressive properties [8], have been verified to contribute to the inactivation of T-cells and antigen presenting cells during various diseases, particularly in cancer [9, 10]. The role of MDSCs in autoimmune diseases is just starting to be elucidated. Recently, studies have identified the pathogenic role of MDSCs in ITP. Impaired MDSCs have been implicated in the pathogenesis of ITP [6] via direct inhibition of T cell activation or the indirect induction of regulatory T cells (Tregs), which could also act as a prognostic marker for treatment responses in ITP [7]. As another important myeloid-derived cell compartment, macrophages have been implicated to be active in the perturbation of immune tolerance in ITP. The utmost emphasis placed on macrophages is the phagocytosis function, especially the disturbed balance of the activating and inhibitory Fcγ receptor (FcγRs) [11]. Alternatively activated, also known as M2 macrophages, which are predominantly characterized as CD68^+^ (pan macrophages marker) and CD163^+^ (M2 specific marker), could dampen the immune response and limit inflammation. It has been argued to be conducive to the progression of neoplasia [12, 13] as well as to successfully inhibit experimental autoimmune encephalomyelitis [14, 15]. However, M2 macrophages have not yet been investigated properly in ITP patients.

In the present study, we intended to determine the features of circulating M2-like macrophages, to examine its relationship with MDSCs, and to evaluate the proinflammatory milieu leading to the expansion of these two cell compartments, thereby providing novel insights for the immune dysregulation as well as prognostic indicators of ITP.

## Methods

### Patients and controls

Thirty-three patients with newly diagnosed primary ITP with blood platelets count less than 30 × 10^9^/L and required medical intervention were enrolled in the study between September 2015 and May 2017. All patients met the diagnosis criteria of ITP according to an international working group [16], cases with diabetes, hypertension, cardiovascular diseases, pregnancy, active or chronic infection, connective tissue diseases were excluded from this study. Bone marrow aspirate was performed to exclude other diseases resulting in thrombocytopenia [17]. All patients received HD-DXM regimen (40 mg of oral dexamethasone daily for 4 consecutive days). A complete response (CR) was defined as an increase in platelet level to ≥ 100 × 10^9^/L by day 10 after the initiation of treatment. Partial response (PR) was defined as platelet counts (30–100 × 10^9^/L) and no response (NR) was defined as a platelet level still < 30 × 10^9^/L by day 10 after treatment. Other treatments were considered if there was no response to dexamethasone. The control group consisted of 18 healthy volunteers. The present study was approved by institutional review board of Zhongshan Hospital Fudan University in accordance with the Declaration of Helsinki, and written informed consent was obtained from all patients.

### Isolation of peripheral blood mononuclear cells

EDTA-anticoagulated venous blood samples were freshly collected from ITP patients before and 10 days after dexamethasone treatment and from healthy volunteers. Peripheral blood mononuclear cells (PBMCs) were isolated by Ficoll density-gradient centrifugation and resuspended in cell freezing medium (10% DMSO in fetal bovine serum) at 5 × 10^5^ cells/mL. The cell suspensions were frozen at − 80 °C and thawed for testing on separate occasions.

### Flow cytometry analysis

MDSCs were defined as CD11b^+^/CD33^+^/HLA-DR^−^. Single cell suspensions were surface stained with APC-conjugated anti-human CD11b (ICRF44; BD Biosciences, San Diego, CA), FITC-conjugated anti-human CD33 (HIM3-4; BD Biosciences) and PerCP-Cy5.5-conjugated anti-human HLA-DR (G46-6; BD Biosciences). All samples were prepared according to the manufactures’ instructions. The phenotype of circulating M2-like macrophages was analyzed by intracellular CD68 (Y1/82A; BD Biosciences) and CD163 (GHI/61; BD Biosciences) staining after fixing with 4% paraformaldehyde and permeabilizing by FACS permeabilizing solution (BD PharMingen, San Diego, CA). Flow analysis was performed on a FACS Aria II flow cytometer (BD Biosciences) and data were analyzed using FlowJo 7.6.1 (Tree Star, San Carlos, CA) software.

### Plasma inflammation cytokine array

Cytokine profiles were measured by Quantibody Human Inflammation Array 3 (RayBiotech, Norcross, GA) that detected 40 inflammation-associated cytokines simultaneously using plasma samples (10 healthy controls and 23 out of these 33 primary ITP patients) cryopreserved at − 80 °C. The array slides were incubated with thawed plasma samples. Then washed and incubated with a cocktail of biotinylated antibodies according to the protocol provided by the manufacturer. The slides bounded with biotin were then incubated with streptavidin-conjugated Hylite Plus 555 fluor. Relative fluorescent strength was measured by LuxScan 10 K-A Microarray Scanner (CapitalBio Corporation, Beijing, China).

### Statistical analysis

All data were expressed as mean ± standard deviation, and were analyzed using the SPSS 13.0 software (SPSS, Chicago, IL). Continuous variables between groups were determined by the one-way ANOVA or Mann–Whitney U test as appropriate. Two-tailed Student’s non-paired *t* test was applied for evaluating statistically significant differences between two independent groups, and the differences between pre- and post-treatment groups were detected by paired Student’s *t* test. The correlation between MDSCs and M2-like macrophages was accessed by Pearson’s correlation coefficient. Two-tailed *p* < 0.05 was considered to be statistically significant (Additional file 1: Figure S1).

## Results

### Clinical characteristics of primary ITP patients

Among 33 ITP patients in the present study, 22 achieved CR with dexamethasone while 5 patients achieved PR and 6 unresponsive patients. Clinical characteristics were presented in Table 1. The median age of eighteen healthy controls (10 females and 8 males) was 44 years (range 34–53 years) and the median platelet count was 236 × 10^9^/L (range 114–304 × 10^9^/L).

**Table 1:** Clinical characteristics of ITP patients

Characteristics	All patients (N = 33)	
	No.	%
Gender		
Female	19/33	58
Male	14/33	42
Age, years median (range)	47 (28, 80)	–
Platelet counts (×10^9^/L), median (range)		
Before treatment	9 (2, 29)	–
After treatment	105 (3, 220)	–
Response to HD-DXM regimen		
CR	22/33	67
PR	5/33	15
NR	6/33	18

### Expansion of peripheral MDSCs but not M2-like macrophages in ITP

**Fig. 1:**
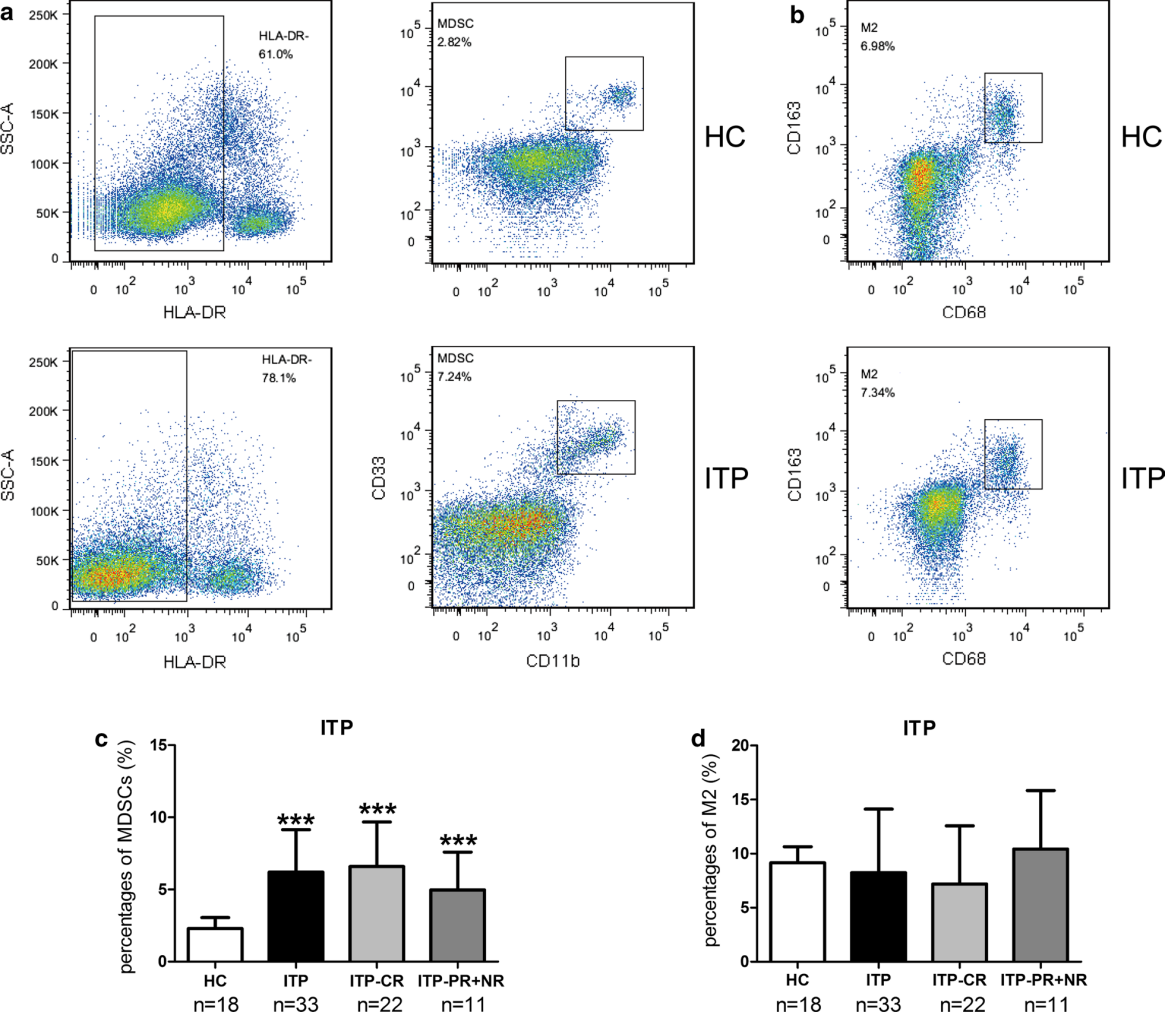
Peripheral MDSCs and M2-like macrophages in newly diagnosed ITP patients (n = 33) before treatment and health control (n = 18). **a** Representative dot plots of CD11b^+^CD33^+^HLA-DR^−^ MDSCs in the PBMCs of HC and ITP patients. **b** Representative dot plots of CD68^+^CD163^+^ M2-like macrophages in the PBMCs of HC and ITP patients. **c** Peripheral MDSCs were significantly elevated in ITP patients, with no respect of their later treatment response to HD-DXM regimen. **d** Peripheral M2-like macrophages were not found elevated in ITP patients, with no respect of their later treatment response to HD-DXM regimen. ***p < 0.001, compared to HC in two-tailed Student’s non-paired *t* test. Bars represented SD

The percentages of MDSCs and M2-like macrophages were analyzed in healthy controls (HC) and ITP patients. Otherwise, ITP patients were further grouped into CR or PR + NR according to their later treatment responses. MDSCs were found significantly elevated in ITP patients comparing with HC (6.21 ± 2.94% vs. 2.29 ± 0.76%, *p* < 0.0001, Fig. 1a, c). When considering treatment response, the expansion of MDSCs was especially accentuated in CR group, but less prominent in PR + NR group (6.60 ± 3.08 vs. 2.29 ± 0.76, CR vs. HC, *p* < 0.0001, and 4.97 ± 2.63 vs. 2.29 ± 0.76, PR + NR vs. HC, *p* = 0.23, Fig. 1c). No significant difference was observed in circulating M2-like macrophages among ITP patients when compared with HC (Fig. 1b, d). These experiments pointed towards the dramatically elevated percentages of MDSCs in ITP patients, especially among the CR group (Additional file 2: Figure S2).

### Peripheral M2-like macrophages correlated positively to MDSCs in ITP

**Fig. 2:**
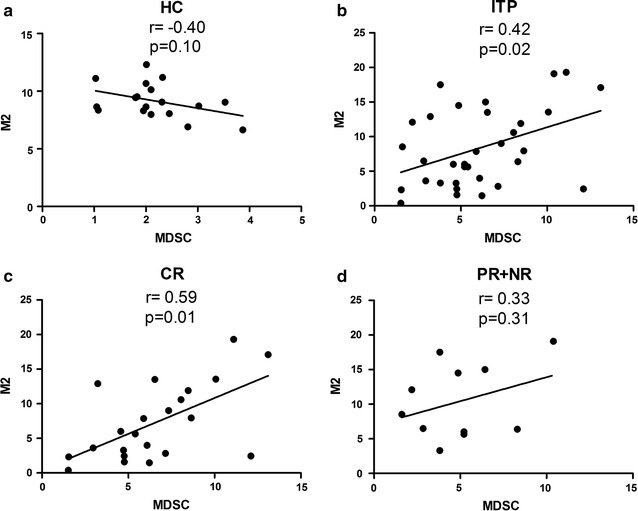
Peripheral MDSCs correlated positively with M2-like macrophages in ITP patients before treatment. Correlation between the percentages of MDSCs and M2-like macrophages in HC (n = 18) (**a**) and newly diagnosed ITP patients (n = 33) (**b**), among CR group (n = 22) (**c**) and among PR + NR group (n = 11) (**d**). Data analyzed by Pearson’s correlation test

No significant correlation between M2-like macrophages and MDSCs was noted among the HC (*r* = − 0.40, *p* = 0.10, Fig. 2a), but a significant positive linear correlation was verified in ITP patients (*r* = 0.42, *p* = 0.02, Fig. 2b), especially in cases of the CR group (*r* = 0.59, *p* = 0.01, Fig. 2c). No correlation was found between M2-like macrophages and MDSCs in PR + NR group (*r* = 0.33, *p* = 0.31, Fig. 2d). The positive correlation between peripheral M2-like macrophages and MDSCs was found in ITP patients, but not in HC.

### M2-like macrophages and MDSCs expanded after the treatment in CR group

**Fig. 3:**
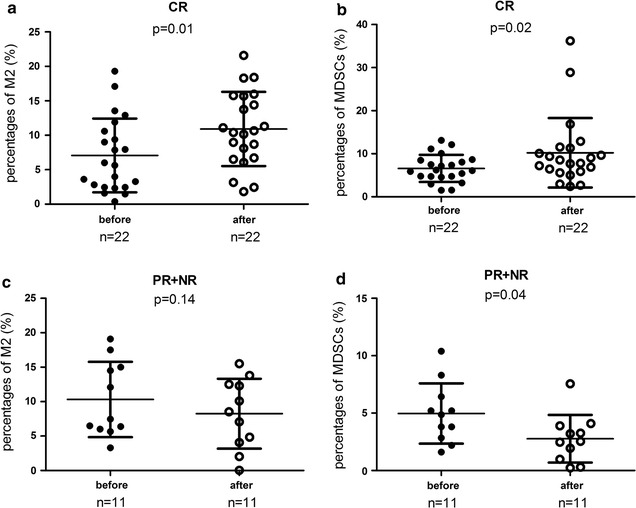
Frequencies of M2-like macrophages and MDSCs increased after treatment among the patients in CR group but decreased in PR + NR group. Differences in M2-like macrophages among the CR group (n = 22) (**a**) and among PR + NR group (n = 11) (**c**); changes in the circulating MDSCs among the CR group (**b**) and among PR + NR group (**d**) monitored before and after treatment. Data analyzed by paired Student’s *t* test

Since all the ITP patients received HD-DXM treatment, we next sought to determine whether it influenced M2-like macrophages or MDSCs. Taking advantages of the matched data, which were taken before and after the treatment of HD-DXM in both CR and PR + NR group, we found the percentage of circulating M2-like macrophages of CR group was significantly elevated after the treatment (7.07 ± 5.35% vs. 10.91 ± 5.39%, *p* = 0.01, Fig. 3a), and so were the MDSCs (6.60 ± 3.15% vs. 10.22 ± 8.07%, *p* = 0.02, Fig. 3b). PR + NR patients manifested moderate decrease in M2-like macrophages (10.32 ± 5.47% vs. 8.26 ± 5.08%, *p* = 0.14, Fig. 3c) but significant decline in circulating MDSCs (4.97 ± 2.63% vs. 2.77 ± 2.07%, *p* = 0.04, Fig. 3d). In conclusion, M2-like macrophages, along with MDSCs, increased significantly after the treatment of HD-DXM in the CR group, while decreased in the PR + NR group (Additional file 3: Table S1).

### Plasma cytokines correlated with M2-like macrophages and MDSCs

**Fig. 4:**
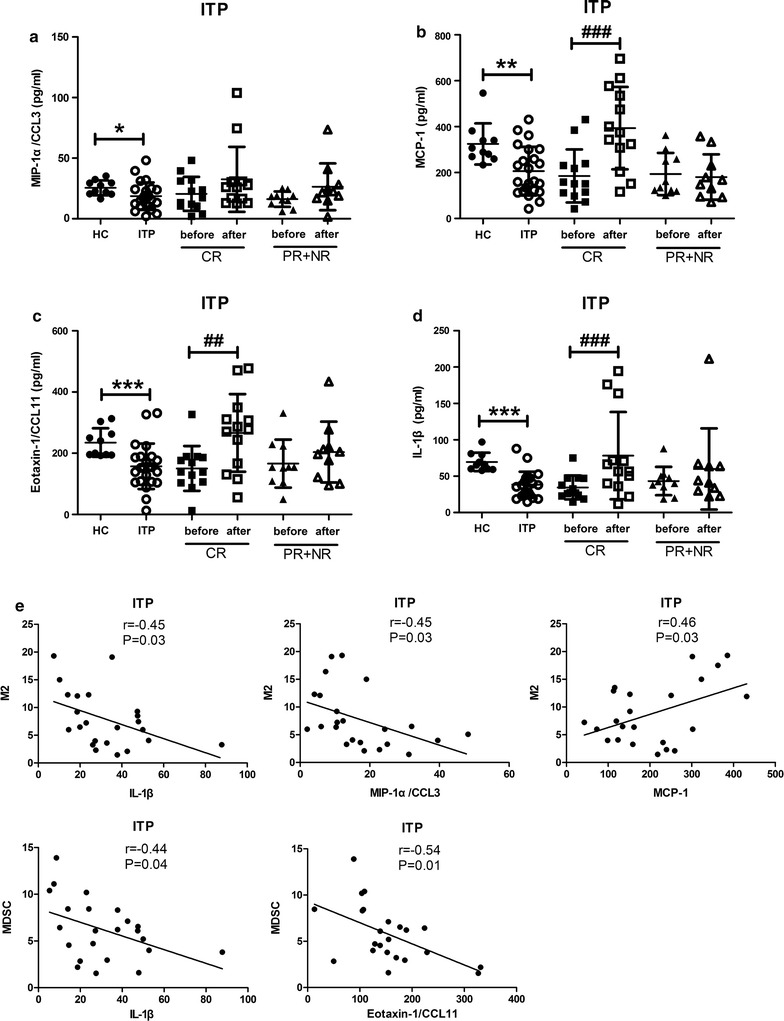
Plasma cytokines correlated with M2-like macrophages and MDSCs. Plasma levels of MIP-1α/CCL3 (**a**), MCP-1 (**b**), Eotaxin-1/CCL11 (**c**) and IL-1β (**d**) in ITP patients. Correlations were found between MDSCs with plasma levels of IL-1β, MIP-1α/CCL3, and MCP-1; between M2-like macrophages with plasma levels of IL-1β and Eotaxin-1/CCL11 (**e**). ***p < 0.001, **p < 0.01, *p < 0.05, ITP (n = 23) compared to HC (n = 10) in two-tailed Mann–Whitney U test; ^###^*p* < 0.001, ^##^*p* < 0.01 after treatment vs. before treatment in corresponding CR group (n = 13) and PR + NR group (n = 10) in two-tailed Mann–Whitney U test

To determine the inflammatory environment of myeloid-derived immune modulator cells, forty inflammation-associated cytokines were analyzed. Within the detection panel of cytokines measured, macrophage-inflammatory protein-1α/CC chemokine ligand 3 (MIP-1α/CCL3), monocyte chemoattractant protein-1 (MCP-1), Eotaxin-1/CeC motif chemokine 11 (CCL11) and interleukin-1β (IL-1β) were found to be significantly down-regulated in primary ITP patients (Table 2, Fig. 4a–d). After treatment, plasma levels of MCP-1, Eotaxin-1/CCL11 and IL-1β were markedly augmented in the CR group (*p* < 0.01, Fig. 4b–d). Correlation analysis between cytokine levels and both myeloid-derived cell concentrations were performed among ITP patients (Fig. 4e). Among all the significantly downregulated cytokines, MIP-1α/CCL3 was found negatively correlated with M2-like macrophages (*r* = − 0.45, *p* = 0.03) while MCP-1 manifested a positive correlation with M2-like macrophages (*r* = 0.46, *p* = 0.03). Eotaxin-1/CCL11 was negatively correlated with MDSCs (*r* = − 0.54, *p* = 0.01). In addition, IL-1β was found to be negatively correlated with both M2-like macrophages (*r* = − 0.45, *p* = 0.03) and MDSCs (*r* = − 0.44, *p* = 0.04). We proposed that low levels of pro-inflammatory cytokines in ITP could provide a moderate inflammatory environment favoring the recruiting and development of M2-like macrophages and MDSCs.

**Table 2:** Plasma cytokines portraits of primary ITP patients (pg/ml)

	Health control (n = 10)	Primary ITP (n = 23)
Eotaxin-1/CCL11	220.40 (195.40–220.40)	154.5 (106.80–189.10)***
Eotaxin-2 (×10^3^)	1.17 (0.84–2.20)	0.92 (0.49–1.41)
G-CSF	825.40 (562.20–855.90)	76.70 (37.00–175.90)***
GM-CSF	80.05 (66.35–97.28)	11.00 (8.00–16.90)***
I-309	83.70 (68.45–134.50)	33.70 (19.60–55.70)***
ICAM-1 (×10^3^)	39.40 (16.75–43.33)	21.87 (6.46–45.08)
IFNγ (×10^3^)	0.79 (0.71–0.94)	0.16 (0.12–0.20)**
IL-1α (×10^3^)	1.16 (0.94–1.33)	0.20 (0.11–0.45)***
IL-1β	65.75 (59.90–79.70)	37.80 (24.10–47.50)***
IL-1ra (×10^3^)	1.28 (1.01–1.63)	0.35 (0.26–0.41)***
IL-2	264.90 (187.80–343.20)	44.90 (33.10–71.60)***
IL-4	415.40 (340.80–504.10)	110.5 (54.7–164.0)***
IL-5	185.40 (158.00–243.40)	42.10 (32.70–51.60)***
IL-6	83.90 (59.03–101.40)	16.50 (7.90–26.80)***
IL-6R (×10^3^)	17.90 (17.86–18.78)	17.80 (17.80–18.90)
IL-7	130.30 (96.93–147.60)	26.20 (22.10–34.60)***
IL-8	99.40 (75.40–121.80)	11.90 (7.70–19.30)***
IL-10	94.45 (63.40–192.20)	17.10 (11.10–23.40)***
IL-11 (×10^3^)	1.43 (1.07–1.93)	0.41 (0.24–0.53)***
IL-12p40	15.85 (12.20–26.78)	13.50 (4.50–18.10)
IL-12p70	41.85 (28.08–55.05)	7.30 (4.60–9.80)***
IL-13	37.80 (26.48–46.33)	10.10 (6.10–12.90)***
IL-15	57.50 (35.35–71.68)	27.60 (12.20–36.90)*
IL-16	124.40 (75.65–171.50)	62.30 (31.80–106.60)*
IL-17A	29.95 (14.50–41.38)	4.00 (1.80–6.80)***
MCP-1 (×10^3^)	0.30 (0.27–0.35)	0.16 (0.12–0.30)**
M-CSF	20.45 (16.28–28.30)	8.20 (4.20–14.00)**
CXCL9/MIG	52.55 (39.88–67.83)	44.60 (25.80–55.00)
MIP-1α/CCL3	25.10 (20.45–30.30)	17.30 (10.60–24.30)*
MIP-1β	97.05 (88.08–140.40)	85.50 (63.50–155.00)
MIP-1d (×10^3^)	1.31 (0.88–2.95)	1.80 (0.95–2.94)
PDGF-BB (×10^3^)	5.36 (3.87–6.72)	0.70 (0.34–1.82)***
RANTES (×10^3^)	14.62 (13.92–15.71)	8.90 (4.08–14.59)**
TIMP-1 (×10^3^)	8.21 (7.00–10.29)	16.13 (11.99–19.03)***
TIMP-2 (×10^3^)	20.46 (13.93–32.76)	11.92 (7.81–14.82)**
TNFα (×10^3^)	1.06 (0.71–1.80)	0.24 (0.15–0.37)***
TNFβ (×10^3^)	0.90 (0.70–1.65)	0.30 (0.21–0.38)***
TNFRI (×10^3^)	5.21 (4.11–5.93)	7.02 (5.40–13.51)*
TNFRII (×10^4^)	1.80 (1.53–2.01)	2.12 (1.82–3.17)*

Data are presented as median (interquartile range)

*CCL11* CeC motif chemokine 11, *G*-*CSF* granulocyte colony-stimulating factor, *GM*-*CSF* granulocyte–macrophage colony-stimulating factor, *ICAM*-*1* intercellular adhesion molecule-1, *IFNγ* interferon-gamma, *IL* interleukin, *MCP*-*1* monocyte chemoattractant protein-1, *M*-*CSF* macrophage colony-stimulating factor, *CXCL9* CXC ligand 9, *MIP* macrophage-inflammatory protein, *PDGF*-*BB* platelet-derived growth factor BB, *RANTES* regulated on activation normal T expressed and secreted chemokines, *TIMP* tissue inhibitor of metalloproteinases, *TNF* tumor necrosis factor, *TNFR* tumor necrosis factor receptor

* p < 0.05; ** p < 0.01; *** p < 0.001, ITP(n = 23) compared with HC(n = 10)

## Discussion

ITP is an acquired autoimmune disease and HD-DXM was adopted as one of the first-line therapy. Antiplatelet autoantibodies and T-cell-mediated cytotoxicity accelerate platelet destruction and impair megakaryocyte maturation in ITP [4, 5]. Abnormal regulatory T cells, and regulatory B cells are involved in disabling immune tolerance in ITP [18–21]. Through examining the percentages of M2-macrophages and MDSCs, their relationship, their further changes after treatment and the cytokine profiles in ITP, our study here provided novel insights into the role of myeloid-derived immune modulator cells in ITP.

Identified as immune modulators, M2 macrophages [15] and MDSCs [8] play active roles in the suppression of autoimmunity. MDSCs are uniformly characterized by the co-expression of myeloid-cell lineage differentiation antigen GR1 and CD11b [22, 23] in mice, but cell surface markers defining human MDSCs have not yet to be confirmed [24–26]. CD11b^+^CD33^+^HLA-DR^−^ cells sharing multiple MDSCs features were recently identified in ITP patients [6, 7]. M2 macrophages are often identified based on the expression patterns of a set of diverse markers. CD163, a cell surface maker, was stained intracellularly in the present study to show the potential monocyte-to-M2 macrophages polarization. Considering the fact that only monocytes but not activated macrophages exist in the peripheral blood, this cell population was defined as M2-like macrophages.

Monocyte-derived macrophages can polarize into pro-inflammatory M1 or anti-inflammatory M2 phenotype in response to various environment stimuli. The preferred M1 polarization in ITP spleens and impaired immunosuppressive expression of M2 markers are involved in the pathogenesis of ITP [27]. MDSCs could expand under pathological conditions including multiple sclerosis [28], rheumatoid arthritis [29, 30] and autoimmune hepatitis [31]. Recently, studies have shown that circulating MDSCs were reduced in ITP patients [6, 7]. In the present study, accumulation of MDSCs but not the M2-like macrophages was observed in ITP patients. Seemingly the accumulated MDSCs might play a protective role to attenuate autoimmunity, but plasma cytokine assay demonstrated a significant downregulation of IL-10, the most important immunosuppressive cytokine secreted by MDSCs that could skew the macrophages polarization towards tumor associated macrophages (TAM) [32]. Thus, the protective effects of MDSCs accumulation may be functionally invalid, which suggests an immediate compensating response to the proinflammatory environment, instead of adequate immunosuppressive properties.

The decreased levels of MIP-1α/CCL3, MCP-1, eotaxin-1/CCL1, and IL-1β, together with other down-regulated pro-inflammatory cytokines produced a moderate proinflammatory environment in ITP. The chemotactic ability of MIP-1α/CCL3 for M2 is significantly stronger than for M1 in spite of the fact that M1 is produced more [33]. In addition to its pro-inflammatory activities, MIP-1α/CCL3 could negatively regulate the proliferation of hematopoietc stem/progenitor cells in leukemia [34]. This presently defined mechanism is likely applicable to elucidate the negative correlation between MIP-1α/CCL3 and M2-like macrophages. MCP-1, a potent proinflammatory chemokine, recruits monocyte/macrophages to sites of inflammation in a wide variety of pathological conditions [35, 36]. An increase in MCP-1 expression has been found to contribute to the M1 macrophages infiltration and to promote tumor rejection, but low to intermediate levels of MCP-1 could recruit M2 macrophages and trigger a profuse vascular network favoring tumor growth [37, 38]. This biphasic action might indicate that the low to mediate level of MCP-1 in ITP might emerge as a potential marker for the recruitment of M2 macrophages.

Eotaxin-1/CCL11 has been shown to promote chemotaxis of eosinophils and mast cells by binding to chemokine receptor 3 (CCR3), but its effects on MDSCs recruitment remain unknown [39]. Recent study hypothesized that eotaxin-1/CCL11 recruits MDSCs in pancreatic ductal adenocarcinoma but in the end the fact is proved to be contrary to this hypothesis [40]. Our data indicated that eotaxin-1/CCL11 was negatively correlated with MDSCs, suggesting that the downregulated eotaxin-1/CCL11 might emerge as a potential marker for the recruitment of MDSCs. The most intriguing finding of the present study was the negative correlation of IL-1β with both M2-like macrophages and MDSCs. A few studies have predicted a positive association between IL-1β and MDSCs. IL-1β could stimulate the accumulation of MDSCs in cancer [41] and MDSCs is also argued to play a significant pro-inflammatory role by inducing Th17 development in an IL-1β-dependent manner [30, 42]. In addition, IL-1β initiates innate immunity through binding with the IL-1 receptors [43] and has been further proved to be essential in the pathogenesis and progression of autoimmune disease, including experimental autoimmune encephalomyelitis [44], systemic lupus erythematous [45] and ITP [46]. Thus IL-1β may be related to M2-like macrophages and MDSC, which are all critical compartments of innate immunity. Low to intermediate levels of pro-inflammatory cytokines in ITP patients might contribute to the construct of a moderate inflammatory environment which favoring the recruitment of both M2-like macrophages and MDSCs, implicating their integrated roles as myeloid-derived immune modulator cells in ITP.

After HD-DXM treatment, M2-like macrophages increased significantly in the CR group, along with the elevated percentages of MDSCs and the increasing levels of IL-10. While in the PR + NR group, both cells populations decreased and the levels of IL-10 remained unchanged. These phenomena might be related to the glucocorticoid efficacy towards MDSCs and M2-like macrophages, and the improvement of these immune modulators might predict better prognosis and explain why some patients just achieved PR but not CR. Known as the classical M2 macrophages activator, IL-4 and IL-13 were significantly upregulated after treatment in the CR group. We postulated that they might be related with the increasing M2-like macrophages. An increase in circulating M2-like macrophages and MDSCs might predict better response in ITP patients. When compared with HC, no significant difference of M2-like macrophages was found before treatment. However, the CR group revealed significant elevated level of M2-like macrophages after treatment, indicating that this novel cell subpopulation may be a potential predictor of better treatment response. Similarly, the continuous increasing of MDSCs in CR group furnished evidence that it may be a reliable indicator of the effectiveness of ITP treatment, unveiling its potential role in measuring the response to different treatment regimens.

It has been shown that 1–5% of MDSCs could develop into myeloid-cell colonies and that about one-third of this colony could differentiate into macrophages under appropriate conditions [47]. In the present study, a significant positive linear association between circulating M2-macrophages and MDSCs was observed in ITP patients, but not in the healthy controls. More intriguingly, not only before the treatment, but also after the treatment, circulating M2-like macrophages frequencies correlated positively to the MDSCs, especially among the CR group, suggesting that this positive correlation might be beneficial to the remission of ITP patients. These positive correlations might be resulted from bidirectional interactions or partially due to their similar background patterns during differentiation. Many tumor analyses have verified that chronic inflammation could enhance the interaction between MDSCs and M2 macrophages [32]. Proinflammation cytokines might play an important role in this orchestrating interaction. In the present study, IL-6 and other inflammatory factors including IL-1β, INF-γ and TNF-α were found to be significantly elevated after treatment, thus developing an inflammatory environment and in turn strengthening the relationship between M2-like macrophages and MDSCs, and driving IL-10 accumulation. The ability of M2-like macrophages and MDSCs to promote and reduce inflammation, seemingly contradictory at first look, might be crucial for the fine tune of the human inflammatory microenvironment.

## Conclusions

The present study focused on the correlative observation of M2-like macrophages and MDSCs in ITP. The percentages of circulating MDSCs expand in newly diagnosed ITP patients, although it might just reflect the quick response to the pathologic environment without any immunosuppressive properties. The increase of circulating M2-like macrophages and MDSCs were found to be correlated with complete response, suggesting that it might predict a better prognosis of ITP. In addition, M2-like macrophages positively correlated with MDSCs in ITP patients. The present study also suggested a novel immune therapeutic approach targeting M2 macrophages and MDSCs in the management of ITP, which deserved further investigation.

## Additional files

**Additional file 1: Figure S1.** The FSC × SSC gate of PBMCs for gating MDSCs (A) and M2-like macrophages (B).

**Additional file 2: Figure S2.** Populations of MDSCs and M2-like macrophages in matched patient samples in the CR group (A) and the PR + NR group (B) before and after HD-DXM regimen. Pre-DXM before treatment of HD-DXM; post-DXM: after treatment of HD-DXM.

**Additional file 3: Table S1.** Clinical characteristics of ITP patients.

## Abbreviations

ITP: immune thrombocytopenia
MDSC: myeloid-derived suppressor cells
HD-DXM: high-dose dexamethasone
CCL11: CeC motif chemokine 11
MCP-1: monocyte chemoattractant protein-1
IL-1β: interleukin-1β
PBMCs: peripheral blood mononuclear cells
MIP-1α/CCL3: macrophage-inflammatory protein-1α/CC chemokine ligand 3
HC: health control
CR: complete response
PR: partial response
NR: no response
