# Supplementary figures and images for: Distinct alterations of CD68+CD163+ M2-like macrophages and myeloid-derived suppressor cells in newly diagnosed primary immune thrombocytopenia with or without CR after high-dose dexamethasone treatment
Source: J Transl Med. 2018 Mar 2;16:48. doi: 10.1186/s12967-018-1424-8 (PMC5833082; doi:10.1186/s12967-018-1424-8)

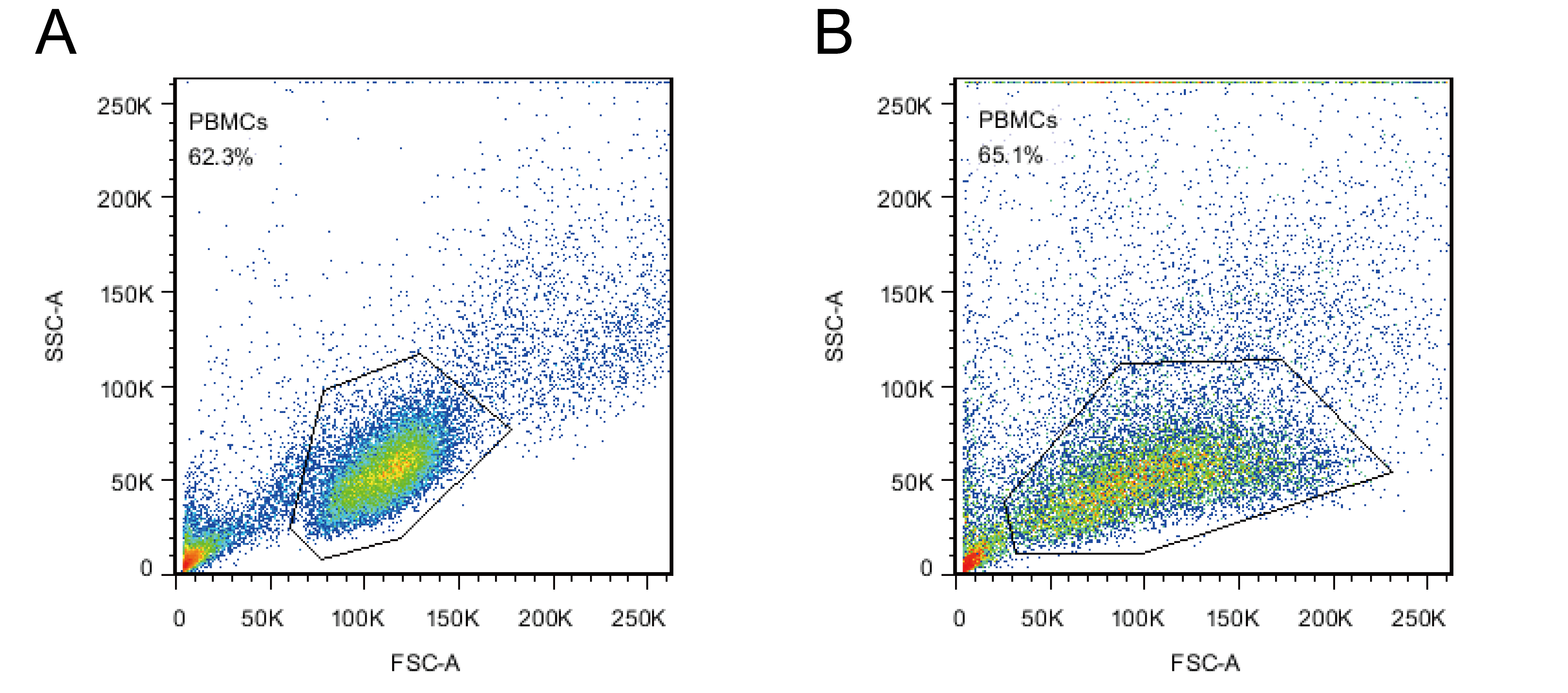

Supplement: Supplementary file 1 — Additional file 1: Figure S1. The FSC × SSC gate of PBMCs for gating MDSCs (A) and M2-like macrophages (B). [file 12967_2018_1424_MOESM1_ESM.tif]

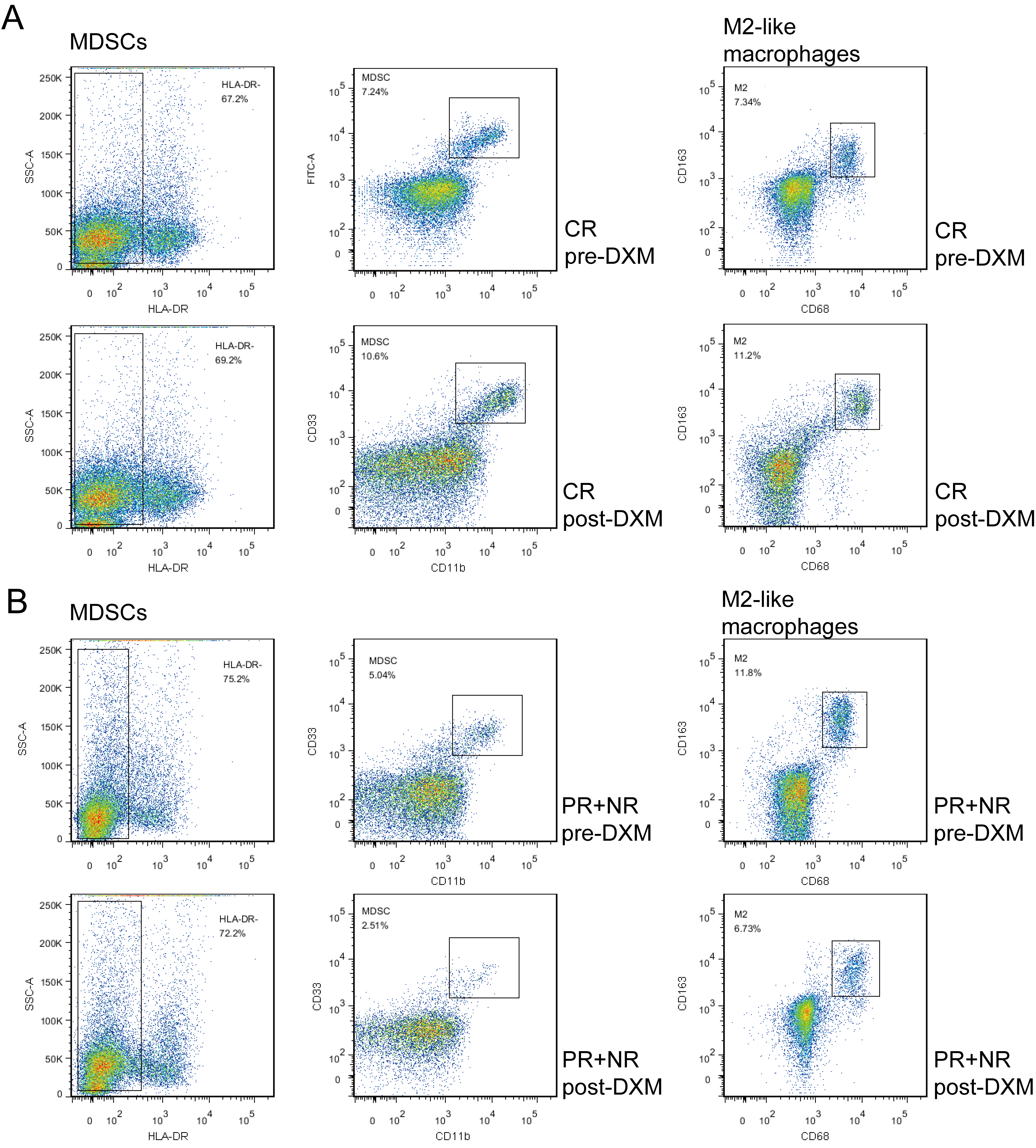

Supplement: Supplementary file 2 — Additional file 2: Figure S2. Populations of MDSCs and M2-like macrophages in matched patient samples in the CR group (A) and the PR + NR group (B) before and after HD-DXM regimen. Pre-DXM before treatment of HD-DXM; post-DXM: after treatment of HD-DXM. [file 12967_2018_1424_MOESM2_ESM.tif]
