# Supplementary material for: Distinct alterations of CD68+CD163+ M2-like macrophages and myeloid-derived suppressor cells in newly diagnosed primary immune thrombocytopenia with or without CR after high-dose dexamethasone treatment
Source: J Transl Med. 2018 Mar 2;16:48. doi: 10.1186/s12967-018-1424-8 (PMC5833082; doi:10.1186/s12967-018-1424-8)
Supplement: Supplementary file 3 — Additional file 3: Table S1. Clinical characteristics of ITP patients. [file 12967_2018_1424_MOESM3_ESM.docx]

**Table S1 Clinical characteristics of ITP patients**

| Patient number | Gender | Age (years) | Platelet counts (×10^9^/L) | | Response to first-line therapies |
| --- | --- | --- | --- | --- | --- |
|  |  |  | Befor treatment | After treatment |  |
| 1 | M | 61 | 3 | 8 | NR |
| 2 | F | 46 | 13 | 15 | NR |
| 3 | M | 39 | 17 | 112 | CR |
| 4 | F | 28 | 14 | 134 | CR |
| 5 | F | 69 | 20 | 165 | CR |
| 6 | M | 33 | 5 | 42 | PR |
| 7 | M | 71 | 29 | 126 | CR |
| 8 | F | 80 | 12 | 152 | CR |
| 9 | F | 72 | 21 | 16 | NR |
| 10 | F | 47 | 5 | 35 | PR |
| 11 | M | 62 | 14 | 76 | PR |
| 12 | F | 67 | 5 | 220 | CR |
| 13 | M | 57 | 7 | 105 | CR |
| 14 | F | 43 | 9 | 103 | CR |
| 15 | F | 52 | 5 | 145 | CR |
| 16 | F | 46 | 8 | 122 | CR |
| 17 | F | 68 | 9 | 200 | CR |
| 18 | F | 23 | 4 | 105 | CR |
| 19 | F | 34 | 9 | 157 | CR |
| 20 | M | 68 | 20 | 175 | CR |
| 21 | M | 59 | 17 | 102 | CR |
| 22 | M | 63 | 8 | 13 | NR |
| 23 | F | 32 | 4 | 45 | PR |
| 24 | M | 44 | 20 | 22 | NR |
| 25 | F | 35 | 2 | 3 | NR |
| 26 | M | 31 | 8 | 40 | PR |
| 27 | F | 64 | 9 | 154 | CR |
| 28 | M | 76 | 15 | 117 | CR |
| 29 | F | 30 | 5 | 107 | CR |
| 30 | F | 36 | 27 | 168 | CR |
| 31 | F | 37 | 7 | 102 | CR |
| 32 | M | 35 | 21 | 103 | CR |
| 33 | M | 67 | 5 | 197 | CR |
| Percentage of famale (%) | 58 | - | - | - | - |
| CR rate (%) | - | - | - | - | 67 |
| Median | - | 47 | 9 | 105 | - |
| (Min-Max) | - | (28-80) | (2-29) | (3-220) | - |
